# Mutual Influence of Sucralose and Bisphenol A on Biological and Neurobehavioral Action in *Drosophila melanogaster*

**DOI:** 10.3390/ijms27114891

**Published:** 2026-05-28

**Authors:** Natasha Miranda, Volodymyr Tkach, Joana Ferreira, Ana Martins-Bessa, Isabel Gaivão

**Affiliations:** 1 Animal and Veterinary Research Center (CECAV), Associate Laboratory for Animal and Veterinary Sciences (AL4AnimalS), University of Trás-os-Montes and Alto Douro (UTAD), 5000-801 Vila Real, Portugal; al79678@alunos.utad.pt (N.M.); abessa@utad.pt (A.M.-B.); 2 Department of Genetics and Biotechnology (DGB–ECVA), University of Trás-os-Montes and Alto Douro (UTAD), Quinta de Prados, 5001-801 Vila Real, Portugal; joanarita20042004@gmail.com

**Keywords:** food safety, artificial sweeteners: food contact substances, neuromotor changes, genomic integrity, genotoxicity

## Abstract

Bisphenol A is a synthetic compound widely used as a monomer in plastic and epoxy resins, whilst sucralose is an artificial sweetener frequently added to foods and beverages. Both substances can be detected in the environment and in food, making the assessment of their combined effects relevant from a food safety and public health perspective. The aim of this study is to investigate the biological and neurobehavioural implications of simultaneous exposure to BPA and sucralose in *Drosophila melanogaster* in vivo. The flies were distributed into two independent experiments: (BPA at 0.5, 1, 2, and 4 mM with a fixed sucralose molarity of 1.25 mM) and (sucralose at 6.3, 12.6, 25.1, and 50.3 mM with a fixed BPA molarity of 0.5 mM) compared to the control group (standard medium). Prolificacy, body weight, longevity, and negative geotaxis were evaluated in adults, whereas locomotor behavior and DNA integrity (Comet assay) were analyzed in L_3_ larvae neuroblasts. The results revealed that the co-exposure to BPA and sucralose induces a significant reduction in prolificity at 0.5 mM BPA (50% at the highest concentrations), as well as in body weight and lifespan (54 days vs. 89 days in the control group); neurobehavioral tests revealed impaired locomotion, with a decrease in movement from 60 s in the control group to 0.67 s in treated larvae. DNA damage confirmed that exposure increases DNA strand breaks. This study provides pioneering in vivo evidence that BPA and sucralose together suggest combined toxicity leading to significant physiological homeostasis disruption. Future research should utilize genetic models with varying antioxidant defense capabilities to further define the metabolic variations in these combined exposures.

## 1. Introduction

The extensive development of the global food industry over recent decades has been accompanied by the targeted synthesis and widespread use of food additives (either natural or synthetic), as well as by the introduction of innovative food packaging materials and targeted synthesis of food additives [1,2,3,4]. These advances have significantly improved food preservation, conservation, palatability, and shelf life. However, they have also led to an increasing presence of food contact substances (FCS) and synthetic additives’ metabolites and degradation products, capable of diffusing towards food products. As a result, humans and other organisms become chronically exposed to complex mixtures of chemical compounds [5,6,7,8,9,10], atypical for normal food and natural conditions. Even in low doses, their combined biological effects include oxidative, genomic and environmental stress, therefore remaining poorly understood.

Among these compounds, artificial sweeteners and plastic-derived chemicals (either monomers or dopants) have attracted particular attention due to their ubiquity and potential biological activity.

**Figure 1:**
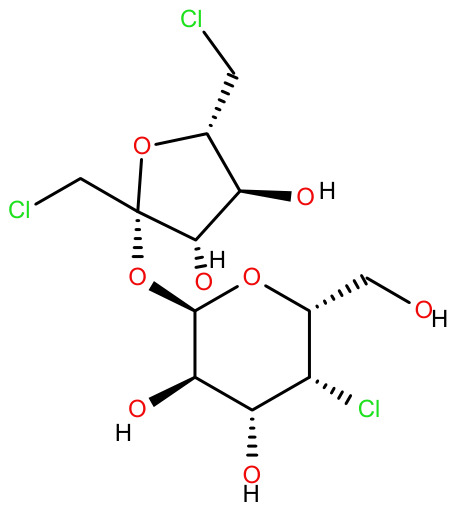
Sucralose chemical structure. Herein, the Oxygen atoms are shown in red, and chlorine atoms, in green.

Sucralose (Figure 1) [11,12,13,14,15] is an artificial sweetener, firstly synthetized in 1976 at Tate & Lyle, alongside with other chlorinated carbohydrates. Its extremely sweet taste was discovered by chance when one of the researchers involved in the synthesis tasted it instead of testing it.

From that time on, its “sweet” history began and even today it is one of the most used sweeteners in the world [16,17,18], as it is 600 to 1000 times sweeter than sucrose (table sugar) and is obtained industrially from it by a three-staged process, including esterification, nucleophilic substitution (yielding 6-acetylsucralose, mentioned below) and ester hydrolysis.

Being a modified carbohydrate derivative, contrarily to aspartame, acesulfame K and saccharin, sucralose possesses an expressively sweet taste, not followed by a bitter aftertaste.

Although it is considered safe for most people to use, its medium- and long-time health and environmental effects are yet to be fully discovered. Some of its negative effects have only been revealed in the last decade and are only now being studied. A recent study involving pregnant and breastfeeding women [19,20,21,22] confirmed that sucralose enters breast milk, causing irreparable damage to the development of the gut microbiota of the human fetus during the last months of pregnancy, as well as in neonates and early-life infants.

Moreover, due to its low biodegradability, sucralose tends to persist and accumulate in the environment [23,24]. Furthermore, when sucralose decomposes thermally or by some bacteria, it transforms into toxic compounds such as dioxins and tetrachlorodibenzofurans. Other issues are the oxidative stresses that sucralose may cause in different organisms and how this can be mitigated [25,26]. Notably, it is imperative to consider the influence of sucralose on a possible induction of oxidative stress and environmental stress in general.

Another critical issue yet to be addressed regarding this compound is the presence of 6-acetyl sucralose, the industrial precursor of the sweetener [27], in reasonable concentrations in industrial product samples. Furthermore, it is likely to appear in the human intestine, where the sucralose is esterified precisely by the hydroxyl group linked to the C6 carbon atom. Recent work has proven the genotoxicity of 6-acetyl sucralose [27,28,29,30].

Toxicological studies of the steric derivative of sucralose proved that the mechanism of its genotoxicity can be considered clastogenic, i.e., it initiates breaks in the DNA structure. Even microscopic concentrations of 6-acetyl sucralose, which can be detected in industrial samples and beverages, exceed the safe threshold of 0.15 μg/person/day. The 6-acetyl sucralose ester increased the expression of genes linked to inflammation, oxidative stress, and carcinogenesis in intestinal epithelial cells, including *MT1G* and *SHMT2*.

Another harmful action of the steric derivative is that it impedes the action of CYP1A2 and CYP2C19, proteins of the cytochrome P450 family, responsible for the transformation of various food substances into a more accessible form, which leads to secondary toxic effects [27,31]. The increase in the genotoxicity of 6-acetyl sucralose concerning sucralose is due to the more significant activity of the secondary organic chloride, linked to the C4 carbon atom (Figure 1), activated by the accepting action of the ester group.

Also, sucralose is the sweetener most frequently used in e-liquids [32,33,34,35,36,37,38], where it is used to hide nicotine and other *N. tabacum* and *N. rustica* alkaloids’ scent and flavor, while also providing vape juice an expressively sweet taste without a bitter aftertaste. Nevertheless, the sucralose metabolites and decomposition products, including the already mentioned 6-acetylsucralose and 6-chlorofructose, might provide additional toxicity to sucralose-containing e-liquid [39,40].

Although initially considered metabolically inert, accumulating evidence suggests that sucralose may interfere with metabolic regulation, gut microbiota composition, and neural signaling pathways. Experimental studies have reported oxidative stress, altered locomotor activity, and behavioral changes following sucralose exposure, raising concerns about its possible neurotoxic and ecotoxic effects, especially under chronic or developmental exposure conditions. Therefore, the study of neurotoxic effects of sucralose alone and in mixtures with other substances (food components, FCS, e-cigarette components, *Diabetes mellitus* drugs, among others) is really up-to-date.

**Figure 2:**
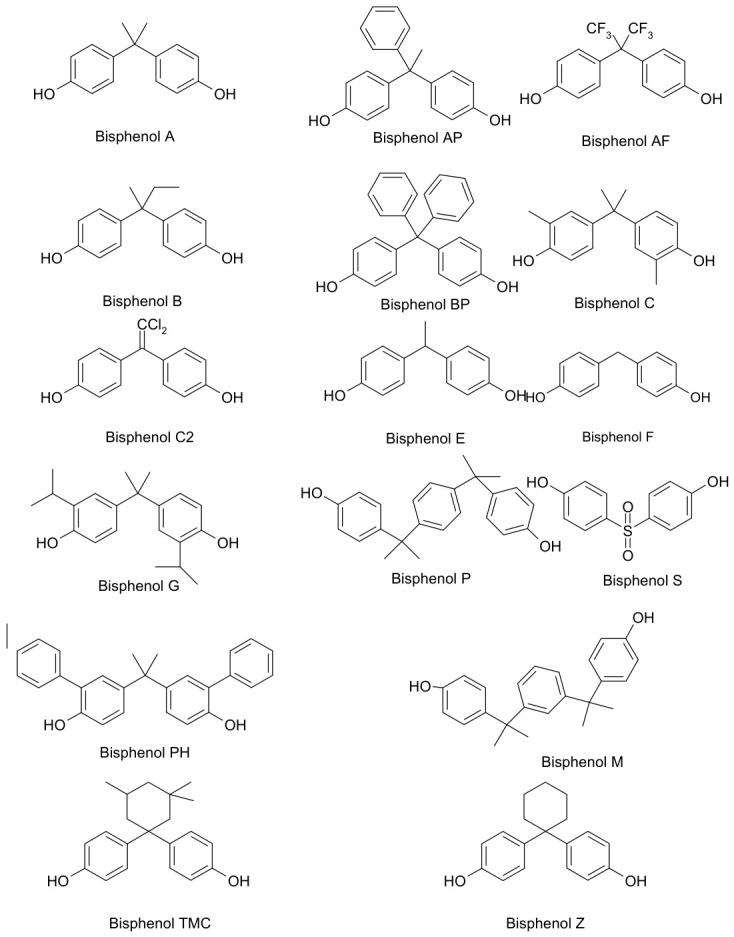
The chemical structures and molecular formulas of the bisphenol family, present in industrial applications and environmental samples.

On the other hand, bisphenols (Figure 2) consist of a group of compounds used mainly in polyester plastics, including polycarbonates and polyphthalates, fast-drying epoxy resin adhesives, and anticorrosion coatings [41,42,43,44,45]. They are also used in odontology for implant fixation, as well as in cosmetical and pharmaceutical formulation packages.

**Figure 3:**
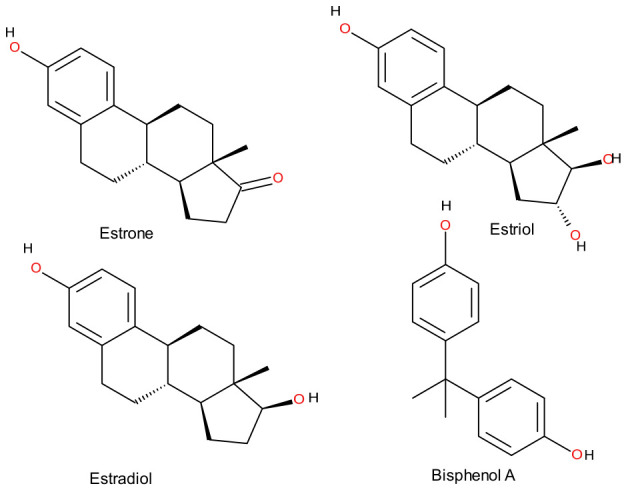
Estrone, estriol, estradiol and BPA with oxygen atoms shown in red.

These compounds are used mostly in polycondensation polymers, which degrade while heated [46,47,48,49,50]. This degradation splits the polymer molecules into monomers, which may penetrate the food or water stored in the polyester ducts or vessels. The main toxic action of bisphenols, principally A, F, and S, consists of endocrine disruptions, mainly xenoestrogenic, acting as an agonist or antagonist through endocrine receptor-dependent signaling pathways, leading, therefore, to several endocrine diseases such as male and female infertility, precocious puberty, various metabolic disorders and hormone-dependent tumors. It also has an active effect on the neuroendocrine system by interfering with neuronal differentiation, the growth and formation of synapses and the individual’s subsequent behavior. The xenoestrogen action of bisphenol is linked to their shape and structural similarity to estrogens–estrone, estriol and estradiol (Figure 3):

The most frequent compound among the class of bisphenols is bisphenol A (BPA), currently one of the most produced compounds worldwide [49,50,51,52,53,54,55,56]. It is often found in food and cosmetic packages and active forms, dental materials, eyeglass lenses, health equipment and thermal paper, among others. Although partially degraded, it accumulates in the environment, being extremely toxic to aquatic organisms [51,52,53,54,55,56]. A recent European Food Safety Authority (EFSA) investigation, done in 2023 and supported by the European Commission, has shown high levels of bisphenol A intoxication in tested people in some EU members, including Portugal [49]. As a result, EFSA banned the use of BPA in food in the European Union since January 2025 [50].

Bisphenol A (BPA) contamination and human intoxication generally occur from exposure to contaminated air (inhalation of contaminated dust, for example), water, effluents and dust, or through the consumption of contaminated food due to leakage of BPA from its containers [57,58]. Another source of BPA intoxication may be e-cigarettes [59,60].

Several inconsistent findings can be found in the literature for this compound, including controversial data concerning a safe level of human exposure to BPA [61]. In 2002, the Scientific Committee on Toxicity, Ecotoxicity and the Environment (CSTEE) concluded that BPA presented a low potential for bioaccumulation, was not corrosive, not mutagenic nor genotoxic, not carcinogenic and a non-inducer of oxidative or membrane damage [62]. However, during its last re-evaluation, the EFSA reduced the tolerable daily dose (TDI) from 50 µg/kg of body weight/day to 4 μg/kg body weight/day [49].

Invertebrate endocrine systems are composed primarily of neuroendocrine components, with true endocrine glands, working in a similar way as vertebrate glands [62]. Insect hormones regulate specific functions such as molting and metamorphosis, yolk synthesis, diuresis, mobilization of fuel for flight, polyphenism and diapause. In *Drosophila,* one of the major hormonal systems involves ecdysteroids, hormones mainly responsible for molting. Molecular studies have demonstrated that the ecdysteroid receptor is homologous to the steroid hormone receptors of vertebrates and that they share a set of conserved features. This suggests that EDCs that can bind to the steroid hormone receptors can also bind to ecdysteroid receptors of invertebrates and possibly interfere with the development, fertility and longevity of exposed individuals [63,64,65]. In fact, BPA has been shown to exhibit anti-ecdysteroid activity in the water flea *Daphnia magna*, causing alterations in the intermoult period and interfering with embryonic development. However, other studies in the literature presented controversial results regarding species’ sensitivity to BPA, showing that, at different relevant concentrations, the effect of BPA differs considerably even between related species and that a lack of clarity about the precise mode of endocrine action remains.

Sucralose and BPA may come into contact through food matrices as a consequence of co-occurrence in processed foods and packaged products, creating realistic exposure scenarios involving chemical mixtures rather than single compounds. Moreover, exposure to them by vaping may also pose danger to the respiratory epithelium.

Such combined exposure is of particular concern because interacting substances may produce additive, synergistic, or antagonistic effects that cannot be predicted from individual toxicity profiles alone. The potential for combined sucralose–BPA exposure to induce genotoxic, neurobehavioral, and ecotoxic effects still remains largely unexplored, especially at biologically relevant concentrations. This exposure may produce either short- or long-term effects.

*D. melanogaster* represents an excellent model organism [66,67,68,69,70] for investigating these combined effects. Its short life cycle, well-characterized genome, conserved molecular and neurobiological pathways, and high sensitivity to environmental toxins make it particularly suitable for toxicological and neurobehavioral studies. Moreover, *Drosophila* allows efficient assessment of locomotor activity, learning, memory, and developmental endpoints, while also enabling mechanistic insights into oxidative stress, DNA damage, and neural dysfunction. These advantages make *D. melanogaster* a powerful and ethically acceptable model for evaluating the exposure of sucralose and bisphenol A on biological and neurobehavioral outcomes.

For this reason, the aim of the present work is to investigate the neurotoxic, genotoxic and ecotoxic potential of sucralose–BPA mixtures, as well as their transgenerational influence on *D. melanogaster*. The present investigation explores the combined impact of these substances, providing novel insights into the negative biological consequences of their simultaneous exposure.

## 2. Results

### 2.1. Prolificacy Evaluation

To evaluate the interaction between BPA and sucralose in the development of *D. melanogaster*, the prolificacy results were contextualized against previous studies with the compounds in isolation (Table 1). This is essential in order to determine whether the mixture induces reproductive and developmental impairment to a greater extent than the compounds individually. Therefore, we align our current findings with the profiles already established by Gaivão et al. [71] and Miranda et al. [72], authors of the present study and laboratory collaborators.

**Figure 4:**
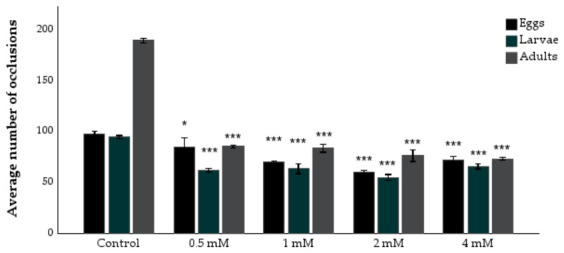
Prolificacy shows the average number of eggs, larvae, and adults obtained from *D. melanogaster* cultures at BPA concentrations of 0.5 mM, 1 mM, 2 mM, and 4 mM with the addition of 1.25 mM of sucralose (n = 20 flies per treatment group, total n = 60 flies). Values represent the mean ± standard deviation (SD) of three independent replicates. Statistical analysis was performed using one-way ANOVA followed by Dunnett’s post hoc test, * *p* < 0.05 and *** *p* < 0.001, with a 95% confidence interval (CI).

**Figure 5:**
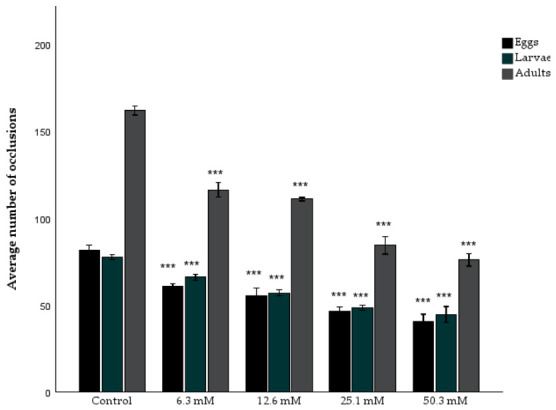
Prolificacy shows the average number of eggs, larvae, and adults obtained from *D. melanogaster* cultures at concentrations of sucralose 6.3 mM, 12.6 mM, 25.1 mM, and 50.3 mM with the addition of BPA 0.5 mM (n = 20 flies per treatment group, total n = 60 flies). Values represent the mean ± standard deviation (SD) of three independent replicates. Statistical analysis was performed using one-way ANOVA followed by Dunnett’s post hoc test, *** *p* < 0.001, with a 95% confidence interval (CI).

The prolificacy results of the two experimental groups exposed to BPA and sucralose are illustrated in Figure 4 and Figure 5. Ten males and ten females were used for mating. A total of 15 vials were used for each concentration, which were conducted in three independent biological assays.

The fertility analysis (Figure 4) revealed that simultaneous exposure to BPA and 1.25 mM of sucralose exerted a detrimental effect on the reproductive capacity in *D. melanogaster*, a significant reduction in eggs at a concentration of 0.5 mM (*p* = 0.010) followed by a dose-dependent decrease at 1, 2, and 4 mM (*p* < 0.001). The impact on larval development and adult emergence was markedly pronounced, with a decline across all tested concentrations compared to the control group (*p* < 0.001). When compared with BPA alone (Table 1), doses of 0.5 and 1 mM did not significantly impair adult development [71], and the addition of a low dose of sucralose (1.25 mM) in the present study was able to induce a significant rate of development. These findings indicate that the combined exposure to BPA–sucralose significantly impairs offspring development.

The assessment of reproduction capacity exhibited a significant detrimental effect of the tested exposures (Figure 5). When comparing the mixtures with the control group, there is a marked decrease in the number of offspring across all concentrations tested (*p* < 0.001). The lowest concentration (6.3 mM sucralose supplemented with 0.5 mM BPA) was sufficient to compromise reproductive success, these findings become relevant when compared to previous observations made by our earlier work for exposure of the compounds in isolation (Table 1), where 0.5 mM of BPA [71] or 6.3 mM of sucralose [72] did not cause a drastic reduction in prolificacy on their own, indicating that the combined exposure disrupts physiological homeostasis more severely than individual exposures to each compound and that combined exposure may cause physiological homeostasis more drastically than individual exposures to each compound, which may lead to a potential synergistic or additive effect between BPA and sucralose. This reduction in prolificacy, sustained up to a dose of 50.3 mM (*p* < 0.001), suggests that the exposure to BPA and sucralose induces a state of systemic physiological stress. This outcome may result from direct interference with vitellogenesis or from early embryonic lethality, in which metabolic resources are likely reallocated toward cellular maintenance and survival mechanisms at the expense of reproductive output.

### 2.2. Body Weight Analysis

**Figure 6:**
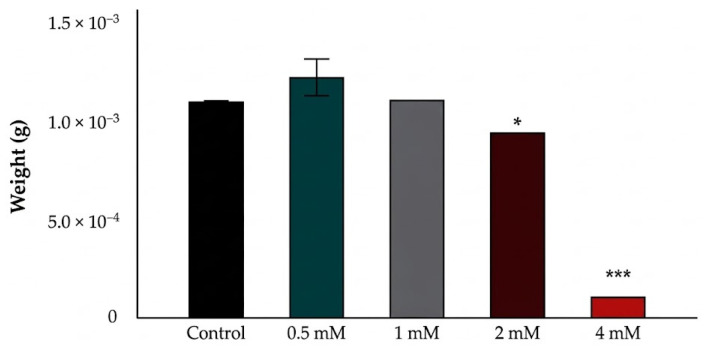
Body weight (g) of *D. melanogaster*, at concentrations of BPA 0.5 mM, 1 mM, 2 mM, and 4 mM with the addition of 1.25 mM of sucralose (n = 100 flies per treatment group, total n = 300 flies). Values represent the mean ± standard deviation (SD) of three independent replicates. Statistical analysis was performed using one-way ANOVA followed by Dunnett’s post hoc test, * *p* < 0.05 and *** *p* < 0.001, with a 95% confidence interval (CI).

**Figure 7:**
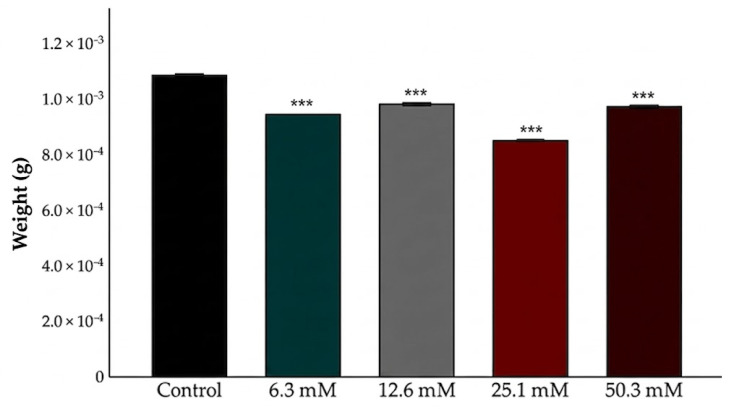
Body weight (g) of *D. melanogaster*, at sucralose concentrations of sucralose 6.3 mM, 12.6 mM, 25.1 mM, and 50.3 mM with the addition of BPA 0.5 mM (n = 100 flies per treatment group, total n = 300 flies). Values represent the mean ± standard deviation (SD) of three independent replicates. Statistical analysis was performed using one-way ANOVA followed by Dunnett’s post hoc test, *** *p* < 0.001, with a 95% confidence interval (CI).

The body mass of *D. melanogaster* exposed to the combination BPA and sucralose was quantified in experimental adults (Figure 6 and Figure 7). Groups of 100 flies per treatment were weighed in triplicate, enabling the estimation of the average individual weight.

The results for adult body mass (Figure 6) revealed an effect dependent on the concentrations studied, with no significant changes observed in fly weight at doses of 0.5 mM and 1 mM BPA (*p* > 0.05). However, in the 2 mM (*p* = 0.028) and 4 mM (*p* < 0.001) tests, a reduction in body mass was observed. Although body weight has not been addressed in individual studies on BPA and sucralose [71,72], current results suggest that concentrations of the BPA–sucralose mixture impair the biomass of *D. melanogaster*. This weight loss at higher doses may reflect nutrient conversion or systemic toxicity that exceeds the organism’s capacity, making it an interesting factor for the present study.

The body mass results (Figure 7) revealed a significant impact on fly mass in all treatments; the exposure to BPA and different sucralose concentrations induced a significant decrease in average individual weight compared to the control group $1.09\times 10^{-3}$ g. At all doses tested (6.3, 12.6, 25.1, and 50.3 mM), a decline was observed (*p* < 0.001), with weight reaching its minimum value of $8.53\times 10^{-4}$ g at a concentration of 25.1 mM. These data suggest that even low concentrations of this mixture can compromise the development processes and potentially disrupt the metabolic homeostasis of *D. melanogaster*.

It can be observed that these results indicate an interaction between the compounds; while 0.5 mM of BPA (Figure 6) was insufficient to alter body mass, increased concentrations of sucralose (Figure 7) had a greater impact on fly weight when combined with BPA, suggesting that sucralose may interfere with metabolic pathways or nutrient absorption, making the organisms more sensitive to BPA-induced toxicity.

### 2.3. Longevity Assay

Here we give an analysis of the impact on the average lifespan of *D. melanogaster* (Table 2), previously established for BPA [71] and sucralose [72] individually, as well as the results of the present assessment of these compounds in combination (BPA and sucralose).

**Figure 8:**
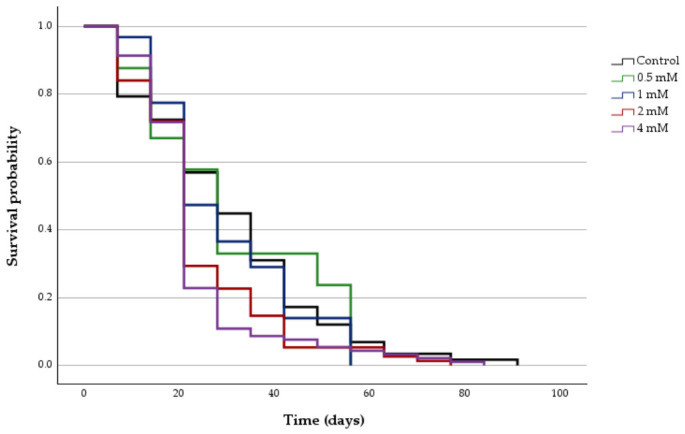
Survival curves of *D. melanogaster* at BPA concentrations of 0.5 mM, 1 mM, 2 mM, and 4 mM BPA with the addition of 1.25 mM of sucralose (n = 120 flies per group). Data analyzed using the Kaplan–Meier method and the Log-rank test: χ^2^ = 15.71, *p* = 0.003, with a 95% confidence interval (CI).

**Figure 9:**
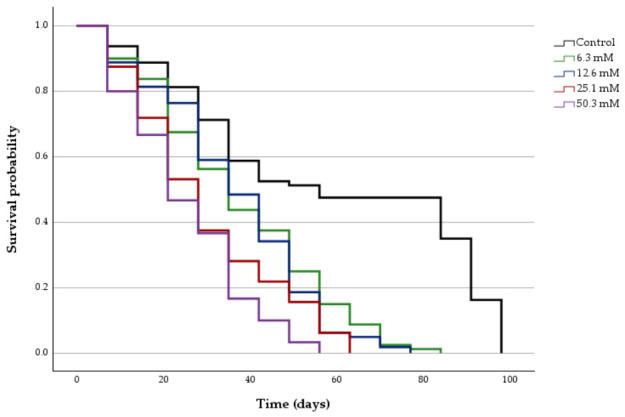
Survival curve of *D. melanogaster* at sucralose concentrations of sucralose 6.3 mM, 12.6 mM, 25.1 mM, and 50.3 mM with the addition of 0.5 mM BPA (n = 120 flies per group). Data using the Kaplan–Meier method and the Log-rank test χ^2^ = 20.33, *p* < 0.001, with a 95% confidence interval (CI).

The Survival curve of *D. melanogaster* following exposure to BPA and sucralose is illustrated in Figure 8 and Figure 9. A total of 15 vials for each concentration, with three independent replicates.

The survival analysis revealed an impact on the survival of flies exposed to the mixture of BPA supplemented with 1.25 mM of sucralose (Figure 8). The control group had a lifespan of 91 days, whereas all concentrations tested induced a significant reduction in life expectancy (*p =* 0.003). At doses of 1 mM, 2 mM and 4 mM, the survival decreased more rapidly, reaching 25% reduction survival compared to the control. although at doses of 0.5 mM the survival was similar to the control. The overall survival curve remained below the control, confirming the deleterious effect of the mixture on adult longevity. These results differ from the data on BPA alone (Table 2), which showed no significant impact on longevity at concentrations of 1.0 mM and 2.0 mM [71], suggesting that the addition of sucralose reduces the toxicological effects of BPA and accelerates mortality.

The results showed that sucralose exposure to sucralose in the presence of 0.5 mM BPA (Figure 9) caused a dose-dependent decrease in the survival of *D. melanogaster.* The control group survived for a maximum of 98 days, and all doses reduced survival (*p* < 0.001). Maximum lifespan was reduced to 84 and 77 days at sucralose concentrations of 6.3 mM and 12.6 mM, respectively. The effect was greater at concentrations of 25.1 mM (63 days) and 50.3 mM (56 days), representing a significant reduction in biological lifespan compared to the control group. Compared to exposure to sucralose alone (Table 2), the average maximum lifespan is typically 112 days [72]; however, the presence of 0.5 mM BPA reduced the average survival time at all tested concentrations. These findings suggest that, in combination with BPA, sucralose may saturate the detoxification pathways, accelerate the aging process, and impact systems homeostasis.

### 2.4. Negative Geotaxis Test

The impact on locomotor performance was assessed using the negative geotaxis test. Table 3 summarizes the results of the tests with sucralose alone [72] and with the mixture of BPA and sucralose. Although previous studies with BPA alone [71] have used behavioral techniques, such as flight activity and social interaction, a comparative analysis of the results will be presented to highlight the specific effects of the interaction of BPA alone.

**Figure 10:**
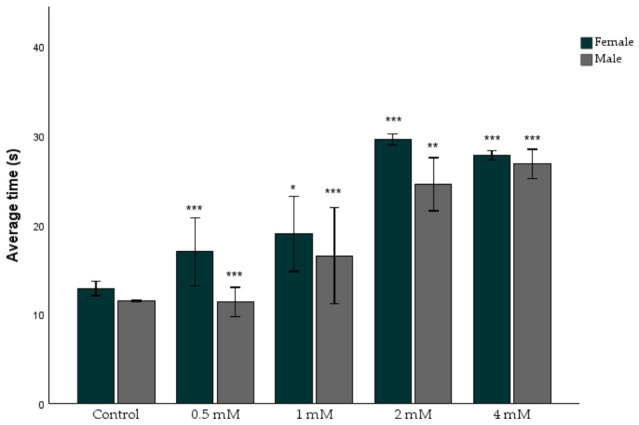
Negative geotaxis of male and female *D. melanogaster* exposed to BPA concentrations of 0.5 mM, 1 mM, 2 mM, and 4 mM BPA with the addition of 1.25 mM of sucralose (n = 20 flies per treatment group total n = 60 flies). Values represent the mean climb time (s) ± standard deviation. Statistical analysis was performed using one-way ANOVA followed by Dunnett’s post hoc test compared to the control * *p* < 0.05; ** *p* < 0.01; *** *p* < 0.001, with a 95% confidence interval (CI).

**Figure 11:**
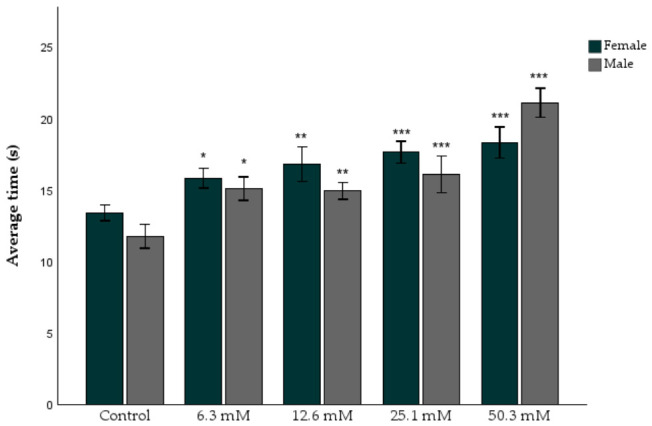
Negative geotaxis of male and female *D. melanogaster* exposed to sucralose concentrations of sucralose 6.3 mM, 12.6 mM, 25.1 mM, and 50.3 mM with the addition of BPA 0.5 mM (n = 20 flies per treatment group total n = 60 flies). Values represent the mean climb time (s) ± standard deviation. Statistical analysis was performed using one-way ANOVA followed by Dunnett’s post hoc test compared to the control, *** *p* < 0.001; ** *p* < 0.01; * *p* < 0.05, with a 95% confidence interval (CI).

Figure 10 and Figure 11 show the time (in seconds) that males and females took to travel an 8 cm distance in the tubes, as well as the respective concentrations of the BPA and sucralose treatments and their respective controls. The analysis was performed independently for each sex, using 15 sample units per group with a total of three independent replicates.

The negative geotaxis test (Figure 10) revealed a significant impairment in the locomotion of *D. melanogaster* in both sexes after exposure to BPA treatment supplemented with of 1.25 mM of sucralose. Both male and female flies showed an increase in climbing time at all tested concentrations (*p* < 0.05). There was a variation according to the doses tested: for females, the effect at 1 mM was less significant (*p* = 0.020) than at higher doses, while for males, the effect at 2 mM was intermediate (*p* = 0.002). However, both sexes displayed a marked motor decline compared to the 4 mM dose (*p* < 0.001), suggesting that high concentrations of the BPA–sucralose mixture have a robust neurotoxic effect that is largely independent of sex. These results, when compared to isolated studies of BPA and sucralose (Table 3), show that the concentrations (1 mM, and 2 mM) did not induce motor deficits, while they did promote hyperactivity [71], indicating that the mixture of compounds significantly increases neuromuscular toxicity.

In the locomotion test (Figure 11), results showed a dose-dependent decline in flies treated with mixture of sucralose at different concentrations of sucralose, 6.3 mM, 12.6 mM, 25.1 mM, and 50.3 mM and 0.5 mM BPA. The climbing time for the control group was recorded as 13.5 s and 11.8 s in females and males, respectively. With increasing concentrations, a significant prolonged time (*p* < 0.001) was recorded. Average time increased to 18.5 s in females and 21.2 s in males treated with 50.3 mM, representing a decrease in motor activity as compared to the control group. Although isolated exposure to sucralose at the highest concentrations induces faster climb times (Table 3), the addition of a dose of BPA (0.5 mM) reversed these data, leading to a significant loss of locomotor coordination.

### 2.5. Isolation and Analysis of the Locomotor Behavior of L_3_ Larvae

**Figure 12:**
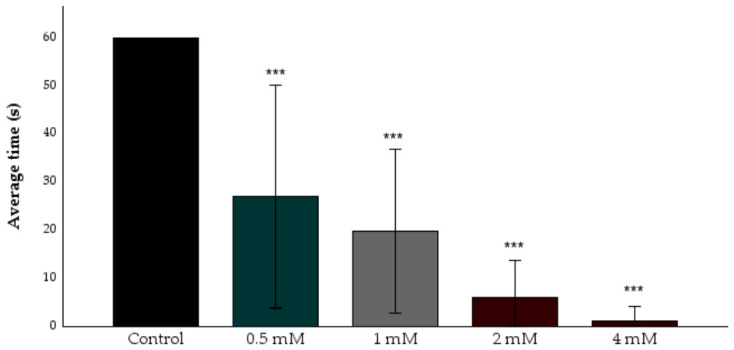
Average active movement time during 60 s of *D. melanogaster* larvae exposed to BPA concentrations of 0.5 mM, 1 mM, 2 mM, and 4 mM BPA with the addition of 1.25 mM sucralose (n = 10 larvae per treatment group total n = 30 larvae). Data represents the mean ± standard deviation. Significant differences were assessed by one-way ANOVA and Dunnett’s post hoc test, *** *p* ≤ 0.001, with a 95% (CI).

**Figure 13:**
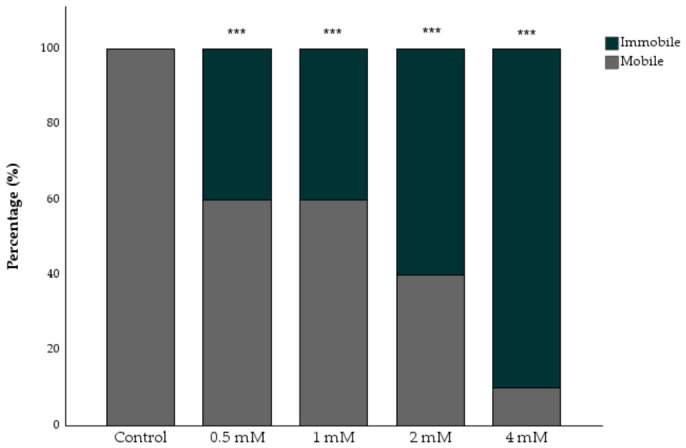
Locomotor behavior of the average number of mobile and immobile larvae in groups of 10 *D. melanogaster* larvae exposed to BPA 0.5 mM, 1 mM, 2 mM and 4 mM with the addition of 1.25 mM of sucralose (n = 10 larvae per treatment group, total n = 30 larvae). Significant differences were assessed by one-way ANOVA and Dunnett’s post hoc test, *** *p* ≤ 0.001, with a 95% CI.

**Figure 14:**
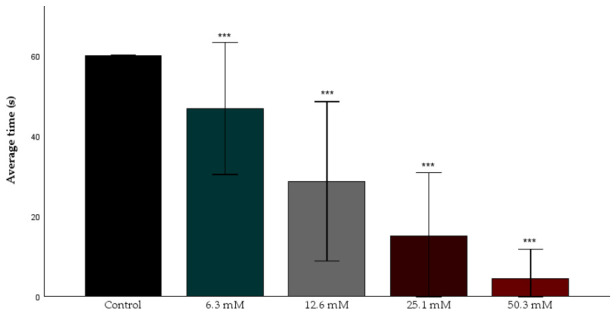
Average active movement time during 60 s of *D. melanogaster* larvae exposed at sucralose concentrations of sucralose 6.3 mM, 12.6 mM, 25.1 mM, and 50.3 mM with the addition of BPA 0.5 mM (n = 10 larvae per treatment group, total n = 30 larvae). Data represents the mean ± standard deviation. Significant differences were assessed by one-way ANOVA and Dunnett’s post hoc test, *** *p* ≤ 0.001, with a 95% (CI).

**Figure 15:**
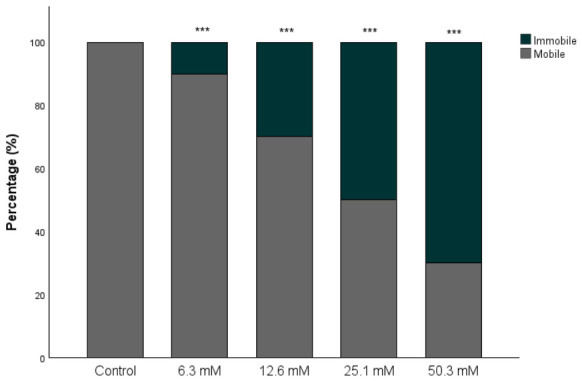
Locomotor behavior of the average number of mobile and immobile larvae in groups of 10 *D. melanogaster* larvae exposed at sucralose concentrations of sucralose 6.3 mM, 12.6 mM, 25.1 mM, and 50.3 mM with the addition of BPA 0.5 mM (n = 10 larvae per treatment group, total n = 30 larvae). Significant differences were assessed by one-way ANOVA and Dunnett’s post hoc test, *** *p* ≤ 0.001, with 95% (CI).

Figure 12 and Figure 13 show the locomotor capacity and viability of *D. melanogaster* larvae in the third instar (L_3_). The analysis shows the average time of active movement during a 1 min interval, while Figure 14 and Figure 15 report the number of mobile and immobile individuals in each group of ten larvae after exposure to BPA and sucralose, with an average of three independent trials compared to their respective controls.

After assessing the activity levels of *D. melanogaster* larvae after exposure to BPA and with the addition of 1.25 mM sucralose (Figure 12), we found that larvae in the control group remained active during the time we observed them, 59.33 s, while those exposed to 4 mM did not move much, remaining active for only 5.33 s, which was a significant difference (*p* < 0.001). However (Figure 13), there was a reduction in overall motility. In the control group, 100% of individuals remained active (mobile). This behavior showed a significant decline at doses of 0.5 mM and 1 mM, with reduced motility of 60%, declining to 40% at a dose of 2 mM. At a concentration of 4 mM, only 10% of the larvae remained active, while 90% reached a state of immobility (*p* < 0.001). These data suggest a neurotoxic effect in the larval stage, which can be characterized by extreme lethargy and functional paralysis. Although larval motility has not been analyzed by isolated studies of compounds, our data suggest that the neurotoxicity of BPA is significantly reduced when combined with sucralose. In isolated studies, BPA concentrations below 1 mM were related to non-significant physiological changes [71]; however, when mixed with 0.5 mM, the given concentrations already induce a 60% reduction in motility, indicating a disruption in larval functional integrity.

Larval motility (L_3_) tests reported that exposure to BPA and increased sucralose compromised neuromuscular integrity in a dose-dependent manner (Figure 14 and Figure 15). In the control group, 100% of the larvae were active, with an average movement time of approximately 59.67 s. In comparison, sucralose concentrations (6.3 to 50.3 mM) in the presence of BPA (0.5 mM) caused a decrease in both assessments (*p* < 0.001). At a dose of 6.3 mM, the average movement time fell to 32.10 s, with immobility of approximately 40%. It was more harmful at doses of 50.3 mM, where the proportion of immobile larvae reached 90% of the sample, and the few individuals that were still able to move did so for 0.67 s (*p* < 0.001). These results reveal a neurotoxic effect compared to isolated exposure to sucralose [72], where behavioral effects were realized in adults, whereas 0.5 mM of BPA, which in isolation is sublethal [71], when combined with sucralose, can lead to almost total larval paralysis, suggesting a potential to degrade resistance and locomotion capacity to a greater extent than the individual forms.

### 2.6. Comet Assay

To analyze the genotoxicity assessment of exposure to BPA and sucralose in isolation [71,72], and the combination of both compounds, a comet assay was performed on *D. melanogaster* neuroblasts, expressed as the percentage of DNA in the tail (Table 4). To achieve linear DNA repair and induce significant DNA breaks, the concentrations (1, 10, 20, 50 mM) in the study with isolated BPA [71] are higher than those in the study with isolated sucralose [72] and the assessments in current studies.

**Figure 16:**
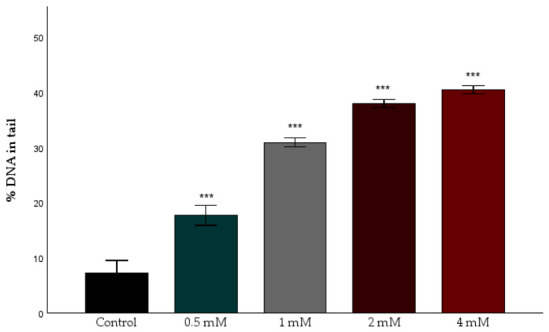
Percentage (%) of DNA in the tail of *D. melanogaster* larvae (L_3_) exposed to BPA concentrations of 0.5 mM, 1 mM, 2 mM, and 4 mM BPA with the addition of 1.25 mM sucralose of three independent biological replicates (n = 100 nuclei per concentration). Statistical significance was assessed using one-way ANOVA followed by Dunnett’s test, *** *p* ≤ 0.001, with a 95% (CI).

**Figure 17:**
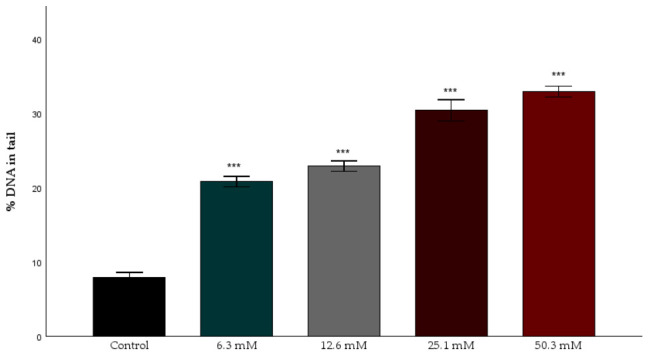
Percentage (%) of DNA in the tail of *D. melanogaster* larvae (L_3_) exposed to sucralose concentrations of sucralose 6.3 mM, 12.6 mM, 25.1 mM, and 50.3 mM with the addition of BPA 0.5 mM of three independent biological replicates (n = 100 nuclei per concentration). Statistical significance was assessed using one-way ANOVA followed by Dunnett’s test, *** *p* ≤ 0.001, with a 95% (CI).

Figure 16 and Figure 17 show the results of the comet assay and the percentage of DNA damage in brain neuroblasts of stage three larvae exposed to BPA and sucralose and their respective controls, tested in three independent replicates.

The results of the comet assay (Figure 16) revealed that the genomic integrity of *D. melanogaster* larvae was compromised by exposure to BPA concentrations and the addition of sucralose. DNA fragmentation increased proportionally to the doses tested: the control group had minimal basal damage, approximately 7.5%, while at a concentration of 0.5 mM, DNA levels in the tail reached approximately 18% (*p* < 0.001). At the highest treatment concentrations of 2 and 4 mM, this damage reached 38% and 41% respectively, suggesting toxicity of cellular repair mechanisms. This may explain the cellular level, early mortality, and motor decline in the adult individuals tested previously.

When comparing the present results with the individual data (Table 4), BPA required high concentrations starting at 10 mM to cause genotoxic damage [71], indicating that the combination of these substances significantly lowers the threshold required to induce DNA fragmentation, even when lower concentrations are used compared to their individual applications.

The percentage assessment of L_3_ larvae neuroblasts revealed that exposure to BPA and sucralose induces a dependent increase in DNA fragmentation (Figure 17). The control group showed reduced basal damage levels, with an average of 7.82% DNA in the tail. Meanwhile, all concentrations tested promoted greater instability than the control (*p* < 0.001). Higher values were observed by increasing doses of 18.45% at the lowest dose (6.3 mM) and reaching a peak of 33% at a concentration of 50.3 mM. These values lead us to note that the mixture of 0.5 mM BPA and increased sucralose in the culture medium has a genotoxic effect, resulting in greater fragmentation of genetic material. In comparison, BPA alone required high concentrations to produce genotoxic effects (Table 4), and the presence of 0.5 mM BPA combined with increasing doses of sucralose was necessary to induce significant DNA damage in all tested groups, highlighting a synergistic genotoxic effect.

## 3. Discussion

Based on the study of the two experiments, it was observed that the exposure of *D. melanogaster* to bisphenol A (BPA) and sucralose compromises physiological homeostasis, revealing a toxic profile that aligns with recent data on non-nutritive sweeteners and endocrine disruptors. In the first group, where BPA concentrations were administered with a fixed amount of sucralose, the reduction in prolificacy, body weight, and longevity, as well as the locomotor decline in larvae and adults and the increase in DNA fragmentation, confirms that BPA, previously characterized as a reproductive and neurotoxicant in *Drosophila* by Musachio et al. [73] and Maria et al. [74], presents significant effects when associated with artificial sweeteners [73,74,75]. These results are consistent with studies by Rani et al. [76], which demonstrated that the combination of BPA with a high-sucrose diet induces a diabetogenic phenotype, tubular dysfunction, and an imbalance of glycemic homeostasis in *Drosophila*, and with Branco et al. [77], who showed that long-term sugar ingestion potentiates the global transcriptomic disruption mediated by BPA, especially in ribosomal and developmental genes. This indicates that nutrient-sensing pathways may decisively modulate BPA toxicity [76,77]. This study indicates that the combination of BPA and sucralose induces toxic effects. In experiment 2, in which sucralose varied in the presence of constant BPA, a pattern was observed where all concentrations of sucralose associated with BPA reduced prolificacy, body weight, and locomotor performance (adult and larval), while significantly inducing genomic instability. This framework reinforces the findings of Miranda et al. [72] as well as those of the in vivo sucralose-sugar study described in the same study, in which a sucralose concentration above 12.6 mM reduced longevity, impaired negative geotaxis, and elevated the percentage of DNA in the tail in *Drosophila*. This is consistent with evidence in mammals that sucralose may promote gut dysbiosis, metabolic inflammation and DNA clastogenesis [72,74]. These results also coincide with reports on the genotoxicity of low-calorie synthetic sweeteners, in which aspartame, acesulfame-K, and saccharin induce an increase in DNA breaks in an in vivo comet assay, suggesting that this group of artificial sweeteners is not biologically inert regarding genomic integrity [78,79].

The association between locomotor decay and genotoxicity observed in the two experimental groups points to oxidative stress and an overload of DNA repair systems in the *Oregon-K* strain, characterized by low antioxidant capacity and efficient activation of the main repair pathways (HR, c-NHEJ, and MMEJ) [80]. While the phenotypic- and genotoxic-level findings are open to interpretation, the literature has established oxidative stress, endocrine dysfunction, and DNA repair overload as biological responses. However, the present study did not directly investigate mechanistic markers such as ROS levels or antioxidant enzymes. Nonetheless, the observed impairment of negative geotaxis in adults and L_3_ larvae motility in both groups is highly compatible with the literature linking BPA to oxidative stress, neurodevelopment alterations damage to dopaminergic neurons, and behavioral phenotypes, as shown by Musachio et al. [73] and Nguyen et al. [81] and with data from Miranda et al. [72] describing impaired locomotion under exposure to sucralose [73,75,81]. Furthermore, the comet assay revealed an increase in the percentage of DNA in the tail for all combined BPA–sucralose treatments, with values at approximately 40% at the highest doses, a pattern that approaches the genotoxicity by oxidative stress described for BPA in *Drosophila* by Anet et al. [82] and Schiffman and Rother [83]. These findings corroborate the case that several synthetic sweeteners show an increase in DNA breaks in comet assays, reinforcing that exposure to sweeteners and endocrine disruptors can, in combination, overwhelm antioxidant defenses and genomic repair systems. Taken together, the results of the two groups indicate that the BPA–sucralose combination reproduces and amplifies toxicity patterns for both BPA in association with sugar and sucralose in association with sugar. When the results of the present study are compared with those of the available literature, it becomes evident that the toxicological risk posed by environmental and dietary mixtures may be underestimated when bisphenol A (BPA), sucralose and other sweeteners are evaluated separately. Studies on *Drosophila* and mammalian models indicate that complex biological responses to these mixtures are mediated by pathways involving oxidative stress, gut microbiota, endocrine signaling and transcriptomic regulation. This has been reviewed by Nguyen et al. [81] and is also evident in recent reviews on sucralose interaction with microbiota, as well as compilations of the genotoxicity of food additives and FCS [81,84,85,86,87,88,89]. Therefore, our findings are in close agreement with those of a recent study by Gaivão [71], which demonstrated that even low-dose exposure to BPA in *Drosophila* triggers significant behavioral changes and molecular stress. The fact that the BPA–sucralose combination amplifies these effects suggests potentiation of the non-monotonic dose–response typical of endocrine disruptors, which could overwhelm the body’s antioxidant defenses and repair systems [71]. Therefore, the present study, by integrating reproductive, metabolic, neurobehavioral, and genomic parameters into two complementary assays (BPA variation with fixed sucralose and sucralose variation with fixed BPA concentration), contributes to consolidating the fact that mixtures of endocrine disruptors and non-nutritive sweeteners can trigger toxic phenotypes, demonstrating the importance of exposure in risk assessment models and the use of *Drosophila* as a model system for human health.

It is important to note that although BPA is the most widely used bisphenol, other members of this chemical group may also exhibit genotoxic properties, particularly given their increasing use in “BPA-free” packaging. Exposure to alternative bisphenols has frequently been assessed at millimolar concentrations. For example, in [90], exposure of *D. melanogaster* to 1 mM bisphenol F (BPF) resulted in impaired regulation of genes crucial for neurodevelopment, including *dFmr1* in *FXS* model strains, as well as other genes associated with synapse formation and neuronal protection. These findings suggest that not only BPA, but also BPF-induced genotoxicity and neurotoxicity, may contribute to developmental delays and autism spectrum disorders.

In [91], Herrero and colleagues exposed the aquatic midge *Chironomus riparius* to the BPA substitute bisphenol S (BPS) at concentrations up to 500 μg/L. BPS exposure led to significant alterations in the expression of genes related to the ecdysone signaling pathway, including *EcR*, *ERR*, *E74*, *cyp18a1*, *hsp70*, *hsp40*, *cyp4g*, *GPx*, and *GST*. These molecular disruptions indicate the potential for major developmental effects in invertebrate models induced by BPS.

Similarly, in [92], several bisphenols—including BPA, BPS, and BPF—were evaluated in *D. melanogaster* for genetically mediated neurotoxic effects. BPA concentrations reached up to 12 mM, while BPF concentrations were as high as 25 mM. Both the nature and severity of neurotoxicity depended strongly on the specific bisphenol tested, with observed toxicity following the order BPZ > BPC and BPAF > BPB > BPS > BPAP ≈ BPA ≈ BPF > BPE. These results highlight the need to reassess the safety of bisphenols proposed as BPA alternatives, particularly those used in BPA-free labeled materials.

At the same time, studies using lower doses of pure BPA and mixtures containing other xenoestrogenic FCS (such as BPS, tetrabromobisphenol A, and bisphenol C2) or synthetic food additives (including sucralose, aspartame, and saccharin) suggest potential enhanced genotoxic effects. Investigating combined actions is essential, as exposure to synthetic compounds may induce mutagenic effects that are not evident when each substance is tested individually.

A recent study by Muhammad Ali [93] evaluates the genotoxicity of bisphenol P (see Figure 2). A range of assays were employed, including the MTT assay, comet assay, micronucleus assay, and real-time PCR for gene expression analysis. Cells were exposed to BPP at concentrations of 0.5, 1, 2, 4, 8, 16, 32, 64, 128, and 256 µM. Treatment with 32 µM BPP, corresponding to the LC_50_, resulted in 50% cell viability after 24 h, as determined by the MTT assay.

The comet assay demonstrated a significant increase in comet tail length in BPP-treated groups compared with controls, indicating enhanced DNA damage. The highest level of DNA damage was observed at the 3 × LC_50_/2 concentration. In the micronucleus assay, the frequency of micronuclei (MNi) exceeded that of binuclei. Additionally, a significantly elevated cytokinesis-block proliferation index (CBPI) was detected at higher BPP concentrations relative to the negative control group. Gene expression analysis revealed increased expression levels of *OGG1* and *HPRT1* in BPP-treated cells compared with untreated controls. Notably, OGG1 expression exhibited a dose-dependent increase, consistent with its role in DNA damage response. Overall, the findings indicate that BPP exerts both cytotoxic and genotoxic effects in MDBK cells. The upregulations of DNA repair genes (*OGG1* and *HPRT1*) may serve as potential biomarkers of BPP-induced genotoxicity. Further investigation into the molecular mechanisms of BPP toxicity and its cross-species effects is warranted to better understand and mitigate its potential harmful impacts.

The study by Ahmad [94] aimed to evaluate the toxicity of BPS by assessing genotoxic, biochemical, histopathological, and oxidative stress parameters in the liver, gills, and kidneys of *Labeo rohita*. Fish were exposed to three concentrations of BPS (400, 800, and 1000 µg/L) for 21 days. A significant (*p* ≤ 0.05) reduction in antioxidant enzyme activities—including superoxide dismutase (SOD), catalase (CAT), reduced glutathione (GSH), and peroxidase (POD)—was observed in all examined tissues. In contrast, oxidative stress markers, such as thiobarbituric acid reactive substances (TBARS) and reactive oxygen species (ROS), were significantly elevated. Comet assay results demonstrated increased olive tail moment values and a higher % of DNA in tail in BPS-exposed groups compared with controls. Histopathological examination using light microscopy revealed multiple structural abnormalities. In the kidneys, these included clustered nuclei, damaged parenchymal cells, expanded sinusoidal spaces, and the presence of melanomacrophages. In the liver, sinusoidal dilation, enlarged hepatic veins, pyknotic nuclei, melanomacrophage accumulation, and cellular necrosis were observed. Gill tissues exhibited bone cell deformities, lamellar aneurysms, hyperplasia, and curvature of secondary lamellae. Hematobiochemical analysis showed significant (*p* ≤ 0.05) increases in hematocrit, white blood cell counts (WBCs), cholesterol, blood glucose, triglycerides, aspartate aminotransferase (AST), alanine aminotransferase (ALT), triiodothyronine (T3), thyroid-stimulating hormone (TSH), thyroxine (T4), urea, and creatinine levels. Conversely, significant decreases were detected in red blood cell counts (RBCs), mean corpuscular hemoglobin (MCH), hemoglobin concentration, and total protein levels. Overall, the findings demonstrate that BPS exerts pronounced toxic effects on the kidneys, gills, and liver of *Labeo rohita*. The compound disrupts normal physiological function by inhibiting antioxidant enzyme activity, inducing oxidative stress and DNA damage, and causing structural alterations in vital organs. These results indicate that BPS poses significant toxicity risks to fish, even at relatively low concentrations, and the presence of sucralose may intensify this action.

For instance, Branco and Lemos [95] examined gene transcription changes in *D. melanogaster* resulting from the exposure to BPA and dietary sugar. Their results demonstrated that sugar intake amplified the biological effects of BPA, supporting the idea that mixtures of BPA and dietary components may contribute to its genotoxicity in the context of the human diet. Considering that sucralose is frequently used instead of or even alongside sugar, this becomes extremely important.

Ha et al. [96] investigated the geno- and neurotoxicity of the bisphenols AP, B, C, C2, E, G, M, P, PH and Z alongside with tetrabromobisphenol A (TBBPA) in *C. elegans* nematode at millimolar concentration. Several bisphenol analogs, most notably BPB, BPC, BPE, and BPG, significantly increased lethality during the embryonic and L1 larval stages. Developmental delays were also observed following exposure to BPAP, BPB, BPC, and BPG, as evidenced by a reduced proportion of individuals reaching adulthood. As for reproductive toxicity, BPAP, BPB, BPC, BPC2, and BPG were found to significantly decrease egg production. Moreover, exposure to these analogs markedly shortened the lifespan of *C. elegans*, particularly in the case of BPAP, BPB, BPC, and BPG, raising concerns about their potential effects on aging processes. Overall, the findings indicate that these bisphenol analogs may exert detrimental effects on development, reproductive capacity, and longevity. For this reason, change in BPA by another bisphenol analog is neither the solution of the problem of BPA toxicity, nor a safe alternative.

Conversely, antioxidant compounds may mitigate BPA toxicity [97]. This study proposes that BPA metabolism leads to the formation of three estrogenic metabolites that disrupt oxidative homeostasis in the human body. Several antioxidants—including sulfur-containing amino acids (taurine), carotenoids (lycopene), polyphenols (curcumin, luteolin, quercetin, naringin, and naringenin), and indolic antioxidants (melatonin)—were shown to inhibit BPA-induced oxidative stress and reduce its neurotoxic and genotoxic effects.

Similarly, Sirasanagandla et al. [98] demonstrated that natural products rich in polyphenolic compounds can attenuate BPA-induced oxidative stress and toxicity. Taken together, these findings indicate that dietary components may either potentiate or alleviate BPA toxicity, depending on their chemical nature and combined biological actions.

As for sucralose, the environmental, genomic and oxidative stress produced by it has only begun to be investigated extensively in the recent decade [99,100,101,102,103]. Moreover, few to no investigations were dedicated to the sucralose toxicity in mixtures with other food additives and environmental contaminants, which becomes important in the context of either food or electronic cigarette consumption or environmental impact.

Nevertheless, the toxic effects of sucralose on different organisms with and without the presence of other substances are manifested by the plurilateral way. Hu et al. [99] demonstrated that sucralose enhances the toxicity of benzo(a)pyrene in mice by inhibiting the P-glycoprotein (PGP)-mediated efflux, thereby promoting intracellular accumulation of the compound, increasing reactive oxygen species (ROS) production, and amplifying cytotoxic effects. Structural alterations in renal tissue were also observed.

The liver and cardiovascular system appear to be particularly vulnerable to sucralose exposure. Wu et al. [100] reported that sucralose exacerbates non-alcoholic fatty liver disease (NAFLD) in mice through activation of the T1R3 receptor, which stimulates ROS generation. Basson et al. [101] further showed that sucralose upregulates PPAR-α, thereby influencing lipid metabolism and energy homeostasis. Consistently, long-term consumption of FDA-approved artificial sweeteners, including sucralose, has been associated with lipid dysregulation and structural alterations in cardiac tissue [102].

Additional evidence of hepatotoxicity was provided by Haq et al. [103], who documented morphological changes in hepatocytes following exposure to artificial sweeteners, with aspartame exhibiting comparatively greater toxicity. El-Haddad [104] reported similar hepatic and renal inflammatory responses, attributing these effects to the pro-oxidant properties of sucralose during chronic exposure.

Neurotoxic effects of sucralose have also been documented in aquatic organisms. In *Daphnia magna*, combined exposure to sucralose and acesulfame induced dose-dependent behavioral and cardiac alterations, alongside with changes in acetylcholinesterase activity [105]. In *Cyprinus carpio*, chronic exposure resulted in DNA damage, elevated ROS levels, and apoptosis in blood cells [106].

Zhang et al. [107] observed that moderate doses of sucralose extended lifespan in *Caenorhabditis elegans*, whereas higher concentrations induced oxidative stress and reduced longevity. In humans, increased sucralose consumption has been associated with neurovascular and cognitive alterations, particularly in individuals with diabetes or obesity, raising concerns regarding its potential role in the development of neurodegenerative disorders.

Further evidence of sucralose’s genotoxic potential has been obtained from plant models. In *Allium cepa*, exposure to aspartame, sorbitol, and sucralose induced various chromosomal abnormalities, notably micronucleus formation during interphase and mitosis in root tip cells—an established marker of genotoxicity [107]. Subsequent studies [108,109] examining sucralose, aspartame, and their combination reported synergistic genotoxic effects in the same model system.

Animal studies have corroborated these findings. Early investigations [110,111] involving prenatal exposure of male Swiss mice to sucralose demonstrated a dose-dependent increase in hematopoietic neoplasms, particularly when sucralose was administered together with its hydrolysis product 6-CF at concentrations of 2000 ppm and 16,000 ppm. These results suggest that sucralose may influence epithelial and glandular tissues in ways promoting both benign and malignant proliferative changes. Therefore, the overall toxicity of sucralose to different organisms and the environment, and also its synergetic impact with other substances, is important.

Importantly, accumulating evidence indicates that the biological effects of sucralose are strongly context-dependent. Bórquez et al. [112] reported alterations in mitochondrial bioenergetics in intestinal cells without a concomitant depletion of ATP, suggesting subtle metabolic modulation rather than overt cytotoxicity. In contrast, Heredia-García et al. [113] and Saad [114] highlighted the potential for joint effects when sucralose is combined with other sweeteners or consumed chronically.

Dietary factors may also influence these outcomes. Elveren [115] and Singh et al. [102,116] proposed that polyphenol-rich diets could mitigate some of the adverse effects associated with sucralose exposure. Moreover, stevia demonstrated comparatively greater anti-inflammatory activity than both sucralose and sucrose [117], underscoring differences in biological impact among commonly used sweeteners.

This opens the door to the rational formulation of sucralose-based beverages enriched with polyphenols. Additionally, some polyphenolic compounds could serve as natural sweeteners, potentially replacing sucralose.

Natural sweeteners and natural-based packages may serve as a sustainable alternative to sucralose and BPA correspondently [118,119,120]. Natural sweeteners, including neohesperetin [118], perillartine [119] and tagatose [120] may serve as effective, safe and sustainable sucralose alternatives. Other sweeteners, like erythritol and stevia, on the other hand, have a dose-related bipolar biological activity.

A circular economic approach to reducing food, lignin and lignocellulose production waste in order to obtain food-compatible, biodegradable packaging is also viable. In [121,122], grape pomace was used to produce sustainable packaging based on polyphenolic compounds with antioxidant properties. Works [123,124] describe using lignin and cellulose production residues to diminish the environmental impact of losing natural compounds, increase waste reuse, and avoid using packages based on environmentally dangerous materials. This favors the substitution of bisphenols with substances that are less harmful to the environment, genome and homeostasis.

The results observed in this study address the combined toxicity of BPA and sucralose by comparing the results of co-exposure with the standardized individual components previously established by our group [71,72]. Molecular investigations, including assessments of mitochondrial dysfunction, antioxidant enzyme activity, and neuronal markers, can also help support the conclusions already established. This leads to the use of the Comet assay, which is included in the current study; it can provide a sensitive measure of DNA damage, serving as a genotoxic indicator that justifies the aforementioned future experiments.

It is essential to note that the concentrations in this study (millimolar) represent a high amount in the diet or the environment. However, while these concentrations in *Drosophila* are necessary for the flies’ detoxification systems and for toxicological parameters in short-term studies, concentrations in the nanomolar or micromolar range to explore the relevance of human environmental exposure may be well evaluated in future studies.

## 4. Materials and Methods

### 4.1. D. melanogaster Strain

The study was conducted using the wild-type strain *Oregon-K* (*OK*). This strain displays an efficient DNA repair capacity for the three mains mechanisms: homologous recombination (HR), classical non-homologous end joining (c-NHEJ), and microhomology-mediated end joining (MMEJ). Moreover, because it exhibits relatively low antioxidant enzyme activity, this biochemically subdued strain makes it well suited for studies addressing oxidative stress-induced DNA damage [80,125].

### 4.2. Culture Medium—Experimental Conditions and Sample Preparation

The flies were housed in climate-controlled chambers at 21 °C and a regulated photo period of 12:12 h (light/dark). The medium formulation, for 1 L of distilled water, consisted of 100 g of sucrose, 100 g of yeast, and 12 g of agar-agar (Labchem, Santo Antão do Tojal, Loures, Portugal), with the addition of 5 mL of propionic acid for microbiological control. Flies were housed in vials sealed with cotton plugs to allow gas exchange and prevent mite contamination. The control group was maintained in this standard culture medium, without the addition of treatments.

All treatments for the experimental groups were administered by incorporating the compounds into the culture medium with distilled water as a solvent. To ensure the chemical integrity of the additives, the compounds were added to the medium only after it had cooled to 60 °C, a temperature below their degradation thresholds. Sucralose is stable up to 119 °C (onset of decomposition), with more advanced decomposition occurring at 140 °C. Bisphenol A (BPA) has a melting point of 150 °C and a boiling point of 252 °C, ensuring its stability during the preparation of the medium. The experiment was based on two independent treatment groups, which were compared with the standard control group:

- Experiment 1: The flies were treated with bisphenol A (BPA) (97%, Acros Organics, Geel, Belgium) at concentrations of 0.5 mM, 1 mM, 2 mM, and 4 mM, each supplemented with a constant concentration of 1.25 mM (0.5 g/L) sucralose (Ukrfarmprom, Kyiv, Ukraine) to the medium.
- Experiment 2: The flies were exposed to sucralose concentrations of 6.3 mM, 12.6 mM, 25.1 mM, and 50.3 mM (corresponding to 0.25%, 0.5%, 1%, and 2%, respectively), each supplemented with a constant concentration of 0.5 mM BPA.

In both experiments, three independent replicates were established per treatment, each containing ten males and virgin females aged between 0 and 3 days. Mating was conducted for 2 to 3 days while the flies were exposed to their respective media (standard or treated) under standardized environmental conditions (21 °C). Once the F_1_ generation emerged, subsequent experimental tests were performed.

The concentrations used in this study were selected based on previous experiments conducted by the same authors of this manuscript for BPA [71], doses of 0.5 mM, 1 mM, 2 mM, and 4 mM were chosen, as they align with the concentrations identified as causing reductions in fertility and longevity in *Drosophila melanogaster*. For sucralose, concentrations of 6.3 mM, 12.6 mM, 25.1 mM, and 50.3 mM (corresponding to 0.25%, 0.5%, 1%, and 2%, respectively) were used, corresponding to levels that induce behavioral and physiological changes [72]. This assay thus allows for a comparative analysis of the effects of isolated exposure versus the effects of exposure to a mixture in the study.

### 4.3. Prolificacy Evaluation

Prolificacy was quantified, using the basal culture medium described in Section 4.2, supplemented with 0.5% activated carbon (Sigma-Aldrich, St. Louis, MO, USA), which was added to both experimental and control groups. For each condition, the numbers of eggs, larvae, and emerging adults were recorded. This addition aimed to increase visual contrast, thereby enhancing the accuracy of egg scoring [72]. All tests were performed in three independent replicates.

### 4.4. Body Weight Analysis

To evaluate the impact of the treatments on the body mass of *D. melanogaster*, flies were subjected to a 2-to-3-day exposure period to BPA and sucralose (Section 4.2). Following exposure, individuals were anesthetized using diethyl ether until immobilized. Groups of 100 flies were randomly selected from each treatment and the control group and weighed using an analytical balance. The average individual weight was determined by dividing the total mass by the number of individuals (n = 100). The assays were conducted in three independent replicates.

### 4.5. Longevity Assay

Upon emergence of the F_1_ generation, adult flies from each experimental group (control and treatments) were housed in vials plugged with cotton containing approximately 25 mL of culture medium, maintained in triplicate. To ensure nutritional consistency stability and prevent generation overlapping, standardized transfer protocol was implemented, moving the flies to fresh culture medium every 7 days. Mortality was monitored and recorded during each transfer until the death of the last individual. Longevity was defined as the total lifespan (in days), calculated from hatching to death.

### 4.6. Negative Geotaxis Test

The locomotor ability was assessed via the negative geotaxis test, as previously described [72,126]. Groups of ten flies, segregated by sex, were placed in 15 cm vertical glass tubes. To induce the geotactic response, the tubes were mechanically stimulated (three taps against a flat surface). The time required for each fly to reach the 8 cm mark was precisely timed to calculate the average climbing speed (cm/s). To avoid physical fatigue, the tests were conducted with 1 min intervals between each attempt. The three repetitions were strictly maintained.

### 4.7. Isolation and Analysis of the Locomotor Behavior of L_3_ Larvae

Locomotor Behavior Third-stage larvae (L_3_) were harvested following experimental treatments. To isolate the larvae from culture debris, a sucrose flotation technique was employed. Larvae were immersed in a 20% (g/v) sucrose solution and homogenized manually for 30 s. This procedure allowed for the sedimentation of debris while the larvae were recovered by buoyancy due to density differentials [127].

#### 4.7.1. Larvae-Washing and Preparation

To remove sucrose traces, larvae were subjected to sequential washing steps. The individuals were transferred to PBS (pH 7.4) and the process was repeated twice to ensure complete removal of impurities prior to behavioral analysis.

#### 4.7.2. Analysis of Locomotor Behavior

Following the washing procedure, larval locomotor activity was assessed. Larvae were placed on a Petri dish containing a nutrient-free agar layer serving as a standardized substrate for movement. A total of 10 larvae per group were recorded for 1 min. Video analysis was utilized to quantify the proportion of mobile vs. immobile larvae and the cumulative movement duration in (s).

### 4.8. Comet Assay

The alkaline comet assay was employed to quantify DNA strand breaks in larval neuroblasts. Triplicate samples of L_3_ larvae per group were selected, and brains were micro-dissected in Ringer’s solution (150 mM NaCl, 35 mM KCl, and 2 mM CaCl_2_; pH = 6.5 with NaOH). The tissues were processed in 1.5 mL microtubes containing 100 μL of insect Ringer’s solution mechanically using a fine needle to fragment the neuroblasts into a single-cell suspension. The resulting mixture was then centrifuged at 300× *g* for 5 min. The resulting cell pellet was resuspended in 140 μL of 1% low-melting-point agarose (LMP) (Pronadisa, Madrid, Spain) at 37 °C. Duplicated 70 μL aliquots were applied to slides pre-coated with 1% normal melting point agarose (Sigma-Aldrich, St. Louis, MO, USA). After 5 min solidification at 4 °C, slides were immersed in lysis buffer (2.5 M NaCl, 0.1 M EDTA, 10 mM Tris-base, 1% Triton X-100, pH 10) for 1 h at 4 °C. DNA denaturation was performed in electrophoresis buffer (0.3 M NaOH, 1 mM EDTA, pH 12.6) for 30 min, followed by electrophoresis at 25 V/cm and 300 mA (8 V/cm) for 20 min at 4 °C. After neutralization in PBS and distilled water, DNA was stained with DAPI (1 μg/mL). Analysis was conducted by visual score using fluorescence microscopy (Olympus BX41, 40×). A total of 100 nuclei were randomly scored per concentration (50 nuclei per gel, analyzed in duplicate), damage was quantified on a scale from 0 (undamaged) to 4 (maximum damage). The total score (Arbitrary Units, AU) ranged from 0 to 400, and results were expressed as the percentage of DNA in the tail, dividing by 4, as recommended by Azqueta (2011) [128].

### 4.9. Data Evaluation and Statistical Analysis

Statistical analysis was performed using SPSS v26 (Statistical Package for the Social Sciences). The normality of data distribution was verified using the Shapiro–Wilk test while the homogeneity of variances was assessed using Levene’s test. A one-way Analysis of Variance (ANOVA) was employed to determine significant differences between treatments and the control group, followed by Dunnett’s post hoc test to control error rates in multiple comparisons. For survival analyses, Kaplan–Meier curves were used, and differences between groups were assessed using the log-rank test, with results presented as chi-square values (χ^2^) and *p*-values. Data is presented as the mean ± standard deviation (SD), with a 95% confidence interval (CI). All assays were performed in independent biological triplicate. The sample sizes (n) are shown in the figure captions. The number of individuals was selected based on protocols for statistical analysis of toxicological results in Drosophila. Statistical significance is indicated in the graphs using asterisks.

## 5. Conclusions

From the investigation of biological and behavior effects of the presence of sucralose and bisphenol A, it was possible to conclude that this combination acts as a systemic stressor, affecting the reproduction, development, locomotion, and DNA integrity of *D. melanogaster* as exposure increases. The link between locomotor decline, lower biological fitness, and increased DNA fragmentation suggests that oxidative stress and the overloading of repair systems are the drivers of this toxicity. Moreover, the combination of BPA with sucralose enhances the neuroendocrinal impact of BPA. Based on these findings, endocrine disruptors and sweeteners should not be considered in isolation in regulatory terms, but rather as mixtures combinations, which require attention in the context of diet. Therefore, future research should use genetic models with different pro- and antioxidant capacities to explore transgenerational effects and metabolic profiles. Besides unveiling the results of the BPA–sucralose in vivo biological coactivity for the first time, they open the door for the investigation of the sucralose influence on the environmental, genomic and oxidative stress of other food and e-cigarette components. These studies should also be extended to vertebrate models and human cell cultures and tissues, in order to apply the implications to human health issues.

## Figures and Tables

**Table 1:** Comparison of the average number of eggs, larvae, and adults emerged in *D. melanogaster*. Data include isolated BPA [71] and isolated sucralose [72], along with the results of experiment I: BPA concentrations of 0.5 mM, 1 mM, 2 mM, and 4 mM with the addition of 1.25 mM sucralose and experiment II: sucralose concentrations of 6.3 mM, 12.6 mM, 25.1 mM, and 50.3 mM with the addition of 0.5 mM BPA. Statistical significance: * *p* < 0.05, ** *p* < 0.01, and *** *p* < 0.001 relative to the respective control of each study; n.r. = not reported.

Treatment	Concentration	Eggs (*n*)	Larvae (*n*)	Adults (*n*)
Bisphenol A (BPA) isolated	Control	n.r.	n.r.	75
	0.5 mM	n.r.	n.r.	66
	1.0 mM	n.r.	n.r.	82
	2.0 mM	n.r.	n.r.	55 *
	5.0 mM	n.r.	n.r.	41 ***
Sucralose isolated	Control	161	139	132
	0.25% (corresponding to 6.3 mM)	51 ***	37 ***	31 **
	0.5% (corresponding to 12.6 mM)	46 ***	49 ***	51 ***
	1.% (corresponding to 25.1 mM)	70 ***	72 ***	70 ***
	2.% (corresponding to 50.3 mM)	34 ***	37 ***	54 ***
Experiment I: BPA and sucralose mixture (current study)	Control	96	94	188
	0.5 mM BPA + 1.25 mM sucralose	83 *	60 ***	84 ***
	1 mM BPA + 1.25 mM sucralose	69 ***	63 ***	83 ***
	2 mM BPA + 1.25 mM sucralose	59 ***	53 ***	76 ***
	4 mM BPA + 1.25 mM sucralose	71 ***	65 ***	72 ***
Experiment II: sucralose and BPA mixture (current study)	Control	82	78	163
	6.3 mM sucralose + 0.5 mM BPA	61 ***	67 ***	117 ***
	12.6 mM sucralose + 0.5 mM BPA	56 ***	57 ***	112 ***
	25.1 mM sucralose + 0.5 mM BPA	46 ***	49 ***	85 ***
	50.3 mM sucralose + 0.5 mM BPA	41 **	44 ***	76 ***

**Table 2:** Comparative analysis of the average survival of *D. melanogaster* exposed to isolated BPA and sucralose compounds and mixtures. Data for the individual compounds were adapted from Gaivão et al. and Miranda et al. [71,72]. Significance levels are indicated relative to the control group in each study. (n.s. = not significant).

Treatment	Concentration	Survival Days (Mean)	Significance (vs. Control)
Bisphenol A (BPA) isolated	Control	32	-
	0.5 mM	34	n.s.
	1.0 mM	33	n.s.
	2.0 mM	33	n.s.
	5.0 mM	22	*p* < 0.005
Sucralose isolated	Control	112	-
	0.25% (corresponding to 6.3 mM)	63	*p* = 0.041
	0.5% (corresponding to 12.6 mM)	66	*p* = 0.008
	1.% (corresponding to 25.1 mM)	52	*p* < 0.001
	2.% (corresponding to 50.3 mM)	63	*p* < 0.0001
Experiment I: BPA and sucralose mixture (current study)	Control	91	-
	0.5 mM BPA + 1.25 mM sucralose	88	n.s.
	1 mM BPA + 1.25 mM sucralose	78	*p* = 0.003
	2 mM BPA + 1.25 mM sucralose	76	*p* = 0.003
	4 mM BPA + 1.25 mM sucralose	72	*p* = 0.003
Experiment II: sucralose and BPA mixture (current study)	Control	98	-
	6.3 mM sucralose + 0.5 mM BPA	84	*p* < 0.001
	12.6 mM sucralose + 0.5 mM BPA	77	*p* < 0.001
	25.1 mM sucralose + 0.5 mM BPA	63	*p* < 0.001
	50.3 mM sucralose + 0.5 mM BPA	56	*p* < 0.001

**Table 3:** Locomotor performance of *D. melanogaster* by average seconds in negative geotaxis tests, data from isolated sucralose [72] and data from the BPA and sucralose mixture; (F) corresponds to females and (M) to males. The significance levels are indicated in relation to the control group of each study. (n.s. = not significant).

Treatment	Concentration	Climbing Time (Average Seconds)	Significance
Sucralose isolated	Control	27.7 (F)/14.7 (M)	-
	0.25% (corresponding to 6.3 mM)	25.9 (F)/14.7 (M)	n.s.
	0.5% (corresponding to 12.6 mM)	26.0 (F)/18.4 (M)	n.s.
	1.% (corresponding to 25.1 mM)	17.8 (F)/11.7 (M)	*p* < 0.05
	2.% (corresponding to 50.3 mM)	18.5 (F)/14.4 (M)	*p* < 0.05
Experiment I: BPA and sucralose mixture (current Sđstudy)	Control	13.1 (F)/11.6 (M)	-
	0.5 mM BPA + 1.25 mM sucralose	17.2 (F)/11.5 (M)	*p* < 0.001/n.s.
	1 mM BPA + 1.25 mM sucralose	19.2 (F)/16.7 (M)	*p* = 0.020/*p* < 0.001
	2 mM BPA + 1.25 mM sucralose	29.8 (F)/24.8 (M)	*p* < 0.001/*p* < 0.01
	4 mM BPA + 1.25 mM sucralose	28.1 (F)/27.1 (M)	*p* < 0.001
Experiment II: sucralose and BPA mixture (current study)	Control	13.5 (F)/11.8 (M)	-
	6.3 mM sucralose + 0.5 mM BPA	15.9 (F)/15.2 (M)	*p* < 0.05
	12.6 mM sucralose + 0.5 mM BPA	16.9 (F)/15.1 (M)	*p* < 0.01
	25.1 mM sucralose + 0.5 mM BPA	17.8 (F)/16.2 (M)	*p* < 0.001
	50.3 mM sucralose + 0.5 mM BPA	18.5 (F)/21.2 (M)	*p* < 0.001

Isolated BPA data: were evaluated through flight and social interaction trials, for the locomotor integrity of isolated BPA [71], thus demonstrating a qualitative basis, which recorded that doses of BPA (0.5–2.0 mM) induced hyperactivity and increased flight activity. As observed in Table 3, the presence of sucralose in the mixture may also alter this behavioral phenotype.

**Table 4:** Genotoxicity assessment based on the percentage of DNA in the tail of *D. melanogaster*. The data refer to the individual compounds BPA and sucralose reported by Gaivão et al. [71] and Miranda et al. [72], as well as the combination of both compounds presented in the present study. Significance levels are indicated relative to the control group in each study (n.s. = not significant).

Treatment	Concentration	Tail DNA (%)	Significance
**Bisphenol A (BPA) isolated**	Control	10.75%	-
	1 mM	26.25%	n.s.
	10 mM	57%	*p* < 0.001
	20 mM	57.5%	*p* < 0.05
	50 mM	53.5%	*p* < 0.001
**Sucralose isolated**	Control	5.9%	-
	0.25% (corresponding to 6.3 mM)	10.5%	n.s.
	0.5% (corresponding to 12.6 mM)	20%	n.s.
	1.% (corresponding to 25.1 mM)	22%	*p* < 0.001
	2.% (corresponding to 50.3 mM)	40%	*p* < 0.001
**Experiment I: BPA and sucralose mixture (current study)**	Control	7.5%	-
	0.5 mM BPA + 1.25 mM sucralose	18%	*p* < 0.001
	1 mM BPA + 1.25 mM sucralose	24.5%	*p* < 0.001
	2 mM BPA + 1.25 mM sucralose	38%	*p* < 0.001
	4 mM BPA + 1.25 mM sucralose	41%	*p* < 0.001
**Experiment II: sucralose and BPA mixture (current study)**	Control	7.82%	-
	6.3 mM sucralose + 0.5 mM BPA	18.45%	*p* < 0.001
	12.6 mM sucralose + 0.5 mM BPA	22%	*p* < 0.001
	25.1 mM sucralose + 0.5 mM BPA	28.3%	*p* < 0.001
	50.3 mM sucralose + 0.5 mM BPA	33%	*p* < 0.001

The study of BPA alone [71] used larval neuroblasts corresponding to the cell samples from Experiments I and II, but required toxicological exposures of 1, 10, 20, and 50 mM to determine the genotoxic effect of each dose, which is standard in isolated genotoxicity assays to induce clear DNA strand breaks, whereas the mixture assays focused on lower, environmentally relevant concentrations, as both studies were conducted by the same laboratory team.

## Data Availability

All the new data obtained in this research may be accessed and explained by contacting the correspondent authors.

## Abbreviations

*OK*	*Oregon K* strain
HR	Homologous recombination
c-NHEJ	Classical non-homologous end joining
MMEJ	Microhomology-mediated end joining
BPA	Bisphenol A
LMP	Low melting point
NMP	Normal melting point
DAPI	4′,6-diamidino-2-phenylindole
